# Therapeutic Effect of an Antibody-Derived Peptide in a *Galleria mellonella* Model of Systemic Candidiasis

**DOI:** 10.3390/ijms222010904

**Published:** 2021-10-09

**Authors:** Emerenziana Ottaviano, Elisa Borghi, Laura Giovati, Monica Falleni, Delfina Tosi, Walter Magliani, Giulia Morace, Stefania Conti, Tecla Ciociola

**Affiliations:** 1 Department of Health Sciences, University of Milan, 20142 Milan, Italy; emerenziana.ottaviano@unimi.it (E.O.); elisa.borghi@unimi.it (E.B.); monica.falleni@unimi.it (M.F.); delfina.tosi@unimi.it (D.T.); giulia.morace@unimi.it (G.M.); 2 Department of Medicine and Surgery, University of Parma, 43126 Parma, Italy; laura.giovati@unipr.it (L.G.); walter.magliani@unipr.it (W.M.); tecla.ciociola@unipr.it (T.C.)

**Keywords:** antibody-derived peptides, antifungal peptides, immunomodulatory peptides, *Galleria mellonella*

## Abstract

The synthetic peptide T11F (TCRVDHRGLTF), with sequence identical to a fragment of the constant region of human IgM, and most of its alanine-substituted derivatives proved to possess a significant candidacidal activity in vitro. In this study, the therapeutic efficacy of T11F, D5A, the derivative most active in vitro, and F11A, characterized by a different conformation, was investigated in *Galleria mellonella* larvae infected with *Candida albicans*. A single injection of F11A and D5A derivatives, in contrast with T11F, led to a significant increase in survival of larvae injected with a lethal inoculum of *C. albicans* cells, in comparison with infected animals treated with saline. Peptide modulation of host immunity upon *C. albicans* infection was determined by hemocyte analysis and larval histology, highlighting a different immune stimulation by the studied peptides. F11A, particularly, was the most active in eliciting nodule formation, melanization and fat body activation, leading to a better control of yeast infection. Overall, the obtained data suggest a double role for F11A, able to simultaneously target the fungus and the host immune system, resulting in a more efficient pathogen clearance.

## 1. Introduction

Fungi can cause a wide range of infections in humans, representing a serious threat to human health. It is estimated that more than one billion people suffer from fungal infections worldwide [1,2,3]. The mortality rate of deep-seated mycoses remains unacceptably high, especially in immunocompromised patients, partially due to the insufficient antifungal armamentarium and the increasingly reported fungal resistance [4,5]. New antifungal therapies are under investigation, including new compounds directed to novel molecular targets, new formulations, associations between conventional antifungal drugs and other agents, and drug repurposing of existing drugs [6,7,8,9,10]. Nevertheless, only a few compounds are currently undergoing clinical trials for invasive fungal infections [11,12,13].

Host defense peptides (HDPs) show a wide spectrum of biological activities and are emerging as a group of promising candidates for overcoming antimicrobial resistance due to their rapid and unique antimicrobial action [14,15,16]. Several reviews have described the antibacterial and antifungal properties of HDPs, their mechanisms of actions and their development for clinical applications [17,18,19,20,21,22,23].

Numerous peptides with antifungal activity, particularly against *Candida* spp., have been described, derived from microorganisms, plants, invertebrate and vertebrate animals [18,19,20,22]. Among them can be mentioned human histatins and cathelicidin LL37; of great interest are also cryptic peptides, i.e., fragments of physiological proteins, as hemoglobin, lactoferrin, salivary mucin 7 and albumin [24,25,26,27,28,29,30,31]. Most of these peptides cause membrane alterations and cell lysis, but multiple mechanisms of action, also involving intracellular targets, have been reported [32,33].

For many years, our research group focused on antibody (Ab)-derived peptides with antifungal activity [34,35]. Among them, the synthetic peptide T11F (TCRVDHRGLTF), derived from the constant region of human IgM antibodies, proved to exert a significant antifungal activity in vitro against pathogenic yeasts, including multidrug resistant isolates, while lacking hemolytic, cytotoxic and genotoxic effects on mammalian cells [36]. T11F derivatives, obtained by substitution of each residue with alanine, showed a variable activity in vitro against *C. albicans* in comparison to the parental peptide. In particular, the replacement of positively charged residues or cysteine caused a significant reduction in candidacidal activity, while substitution of the negatively charged residue, aspartic acid, resulted in increased candidacidal activity [36]. T11F and most of its alanine-substituted derivatives proved to acquire a left-handed 3_1_-helix conformation (PPII helix) in aqueous solution, but phenylalanine substitution (peptide F11A) caused a loss of the organized structure resulting in a random coil conformation [37].

In this work, T11F peptide and two of its derivatives, D5A and F11A, respectively, chosen for the improved in vitro anti-*Candida* activity and the different structural conformation in aqueous solution [36,37], were analyzed for their possible therapeutic activity against *C. albicans* experimental systemic infection in the greater wax moth *Galleria mellonella*. Indeed, *G. mellonella* larvae have emerged as an alternative model host for human pathogens, including fungi [38]. In addition to its wide application for studying microbial virulence, this invertebrate model allows for investigating immune defense responses and tissue pathology, and to evaluate the efficacy of antimicrobial compounds [39].

We investigated both the direct antifungal activity and the immunomodulatory properties of the selected peptides. F11A derivative exhibited a more significant therapeutic effect, compared to D5A, in *G. mellonella* infection by *C. albicans*, likely related to its prominent ability to modulate the host’s immune system.

## 2. Results

### 2.1. Visualisation of Peptides’ Direct Antifungal Effects on C. albicans Cells by Scanning Electron Microscopy (SEM)

**Figure 1:**
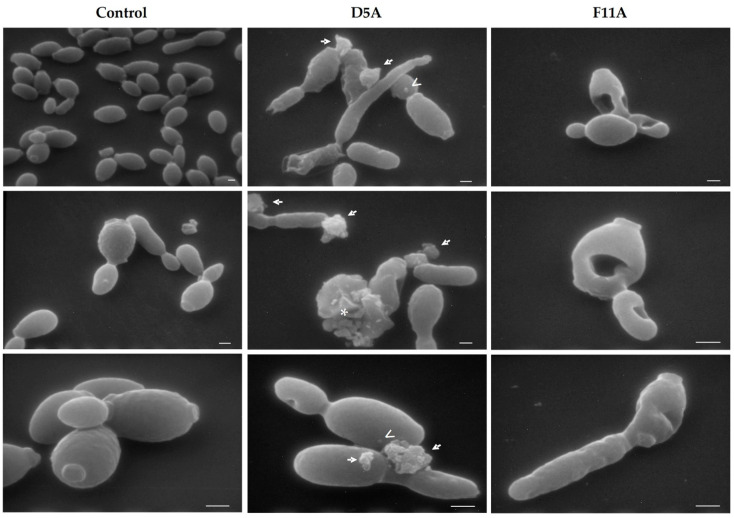
SEM images of *Candida albicans* SC5314 cells treated with D5A and F11A. Structural and morphological alterations were evident after 1 h-treatment with peptides in comparison to untreated (control) cells. Bar = 1 µm. Open arrowheads indicate blebs, arrows indicate the leakage of material from yeast cells and asterisks indicate lysed cells.

In *C. albicans* cells treated with T11F, SEM observation previously showed the presence of blebs and a leakage of cellular material that was also visible by confocal microscopy [37]. Here, we evaluated the direct anti-*Candida* effect of D5A and F11A derivatives. While untreated fungal cells (control) presented a smooth surface and a compact structure (Figure 1, left column), after treatment with D5A the presence of blebs, leakage of cellular material and lysed cells were visible (Figure 1, central column). However, after treatment with F11A gross morphological alterations and deformed fungal cells were visualized, but blebs and leakage of cellular material were not present (Figure 1, right column).

### 2.2. In Vivo Toxicity and Therapeutic Activity of the Investigated Peptides

Toxicity to the host and therapeutic activity against *C. albicans* infection were evaluated for the investigated peptides in *G. mellonella* larvae.

We verified that, under the adopted conditions, there was no significant difference in survival between larvae inoculated with saline (control group) or with the peptides, thus assessing that the peptides were not toxic in this experimental model (Figure S1).

**Figure 2:**
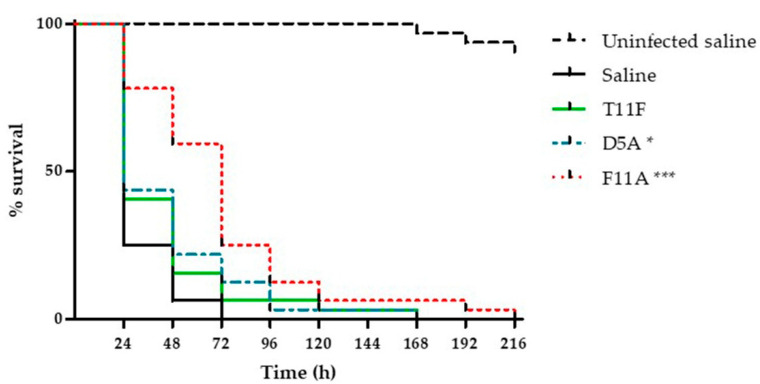
Therapeutic activity of the investigated peptides. *Galleria mellonella* larvae were infected with approximately 5 × 10^5^ cells of *Candida albicans* SC5314 and treated with peptides (10 μmol/kg, single injection 10 μL) or saline (control group). The survival curve of uninfected larvae treated with saline is also shown. The treatment of infected larvae with F11A and D5A significantly increased larvae survival, as assessed by Mantel-Cox log-rank test (* *p* < 0.05; *** *p* < 0.001).

The therapeutic effect was evaluated in larvae infected with a lethal inoculum of *C. albicans* SC5314. Figure 2 reports the pooled results obtained in two independent experiments. A single injection of F11A and, to a lesser extent, of D5A led to a significant increase in survival of larvae in comparison to infected animals treated with saline. T11F was not efficient in improving larval survival. Median survival time was 72 h in F11A-treated group and 24 h in saline-injected larvae (control group), T11F- and D5A-treated groups. All larvae belonging to the control group were dead 72 h post-infection, while, at the same time, 6.25%, 12.5% and 25% of the animals still survived in the groups treated with T11F, D5A and F11A, respectively.

### 2.3. Host Immunomodulation by Investigated Peptides

#### 2.3.1. Hemocyte Analysis

Cellular immune response to fungi in *G. mellonella* involves different hemocytes responsible for pathogen phagocytosis, nodulation and encapsulation (granulocytes and plasmatocytes) with melanin deposition (oenocytoides) [40].

**Figure 3:**
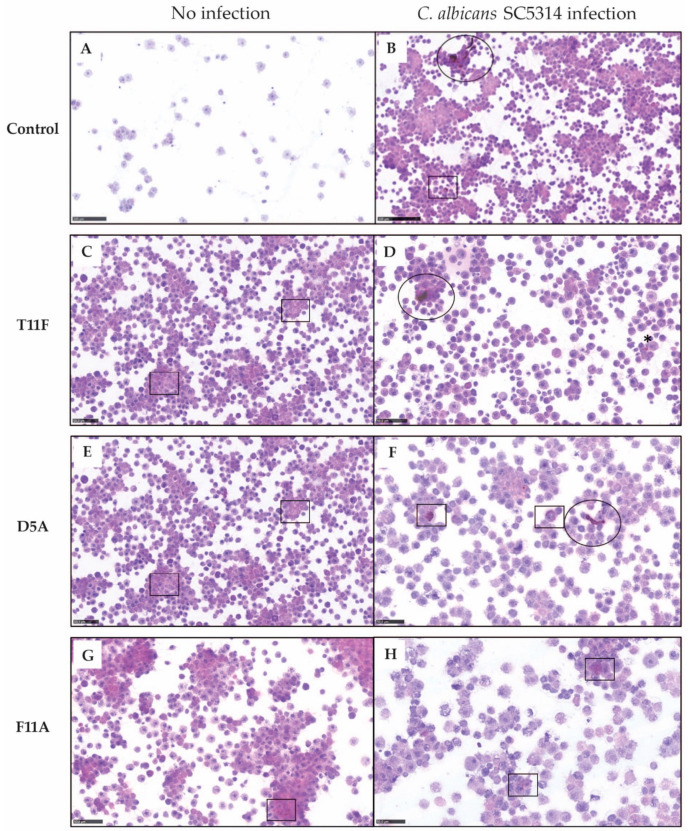
Immunomodulatory activity of the investigated peptides evaluated by cytospin analysis. *Galleria mellonella* larvae uninfected (**A**,**C**,**E**,**G**) or infected with 5 × 10^5^ cells of *Candida albicans* SC5314 (**B**,**D**,**F**,**H**) were treated with peptides (10 μmol/kg, single injection 10 μL) or saline (control group). At 24 h post-inoculation, hemolymph (50 µL) was diluted in saline prior to cyto-centrifugation. Cytospin slides were fixed with Cytofix and stained with hematoxylin and eosin. In control larvae, only a few immune cells were appreciable in the hemolymph (**A**), while *C. albicans* infection elicited hemocyte increase, cell aggregation and melanin deposition (**B**). T11F injection in uninfected larvae (**C**) induced an increased cellularity mostly represented by spherulocytes with no detectable melanization, while in infected larvae (**D**) small melanized aggregates of granulocytes and plasmatocytes around fungal hyphae were observed. D5A (**E**,**F**) effects on larval immune response was highly similar to T11F, with a slight increase of intracytoplasmic melanization. In uninfected F11A-injected larvae (**G**), the hemolymph was rich in hemocytes, with very large clusters composed of centrally-located granulocytes and plasmatocytes surrounded by spherulocytes. Melanization was also detectable in the cytoplasm of immune cells. F11A-treated infected larvae (**H**) showed in the hemolymph a decrease in the number of the immune cells organized in small aggregates with fine intracytoplasmic melanization; fungi were not detectable. Black square: activated immune cells; black circle, dispersed hyphae. Bar = 100 µm (**A**,**B**) and 50 µm (**C**–**H**).

Cytospin analysis performed at 24 h post-inoculation showed a peptide-specific host immunomodulation in both uninfected and infected larvae (Figure 3). In uninfected larvae, the untreated control group (Figure 3A), was characterized by few non-activated hemocytes and the absence of cell aggregates, while treatment with T11F and D5A increased the number of circulating hemocytes, in particular of spherulocytes. Few cell aggregates and, only for D5A, a slight intracytoplasmic melanization of immune cells were observed (Figure 3C,E). F11A led to a strong immune activation. Tridimensional aggregates with central plasmatocytes and granulocytes and peripheral spherulocytes, with weak melanization, were present (Figure 3G).

In larvae infected with *C. albicans* SC5314, treatment with T11F and D5A induced small monodimensional cell aggregates, mainly composed of activated granulocytes and plasmatocytes, and intracytoplasmic weak melanization around fungal hyphae (Figure 3D,F). F11A was more efficient in plasmatocyte and granulocyte activation. Indeed, tridimensional aggregates were visible (Figure 3H). Moreover, neither hyphae nor yeast cells were observed in the hemolymph from F11A-treated larvae. Despite differences in the immune cell recruitment, also the administration of T11F and D54 resulted in a reduction in *C. albicans* filamentous forms in the hemolymph (Figure 3D,F) compared to infected and untreated (control) larvae (Figure 3B).

#### 2.3.2. Larval Histology

**Figure 4:**
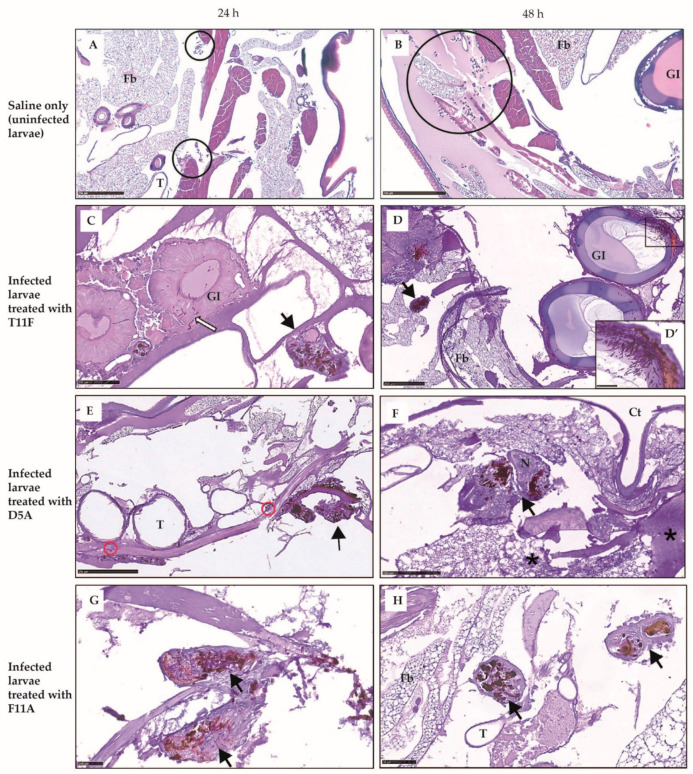
Larval histology of *Candida albicans* SC5314-infected larvae treated with T11F, D5A and F11A peptides. In uninfected larvae injected with saline (control) an activation of the fat body and a progressive increase of circulating hemocytes (circles) were observed (**A**,**B**). *Galleria mellonella* larvae were infected with 5 × 10^5^ fungal cells and treated with peptides (10 μmol/kg, single injection 10 μL). At 24 h post-inoculation, in T11F-treated larvae (**C**) *C. albicans* cells in gastrointestinal lumen and wall (white arrow) and vast areas of melanized cells (black arrow) were visible. At 48 h (**D** and inset **D’**) gastrointestinal tract invasion by filamented, biofilm-organized fungi was observed. At 24 h, D5A-treated larvae (**E**) showed large melanized nodules without signs of encapsulation by hemocytes (black arrow); sparse hyphae were detectable in different tissues (red circles). At 48 h (**F**) a diffuse fungal invasion with small and large melanized nodules in different tissues, including the nervous compartment (black arrow) was shown; heavy irreversible degenerative features of the fat body with hemolymph thickening were also visible (asterisks). At 24 h, in infected larvae treated with F11A (**G**), large melanized nodules around filamentous fungi (black arrow) and activation of the fat body with increase of all immune cell lines were detected; at 48 h (**H**) the infection appeared completely resolved by fungal containment in well encapsulated nodules; the morphology of vital organs seemed to reverse to normal condition. Ct: cuticle; Fb: fat body; GI: gastrointestinal tract; N: neuronal system; T: trachea. Bar = 250 µm (**A**–**F**); Bar = 100 µm (**C**,**G**); Bar = 50 µm (**H** and inset **D’**).

To gain insight into the diverse protective effect observed in vivo, infected larvae treated with different peptides or uninfected larvae treated with saline (control) were processed for histology. In control larvae tissues were well preserved; at 24 h the fat body showed activation and a slight increase in circulating hemocytes was observed (Figure 4A), while at 48 h a further increase of circulating hemocytes released from the activated fat body was seen (Figure 4B). Infected, F11A-treated larvae showed stronger hemocyte recruitment at tissue level, with the fat body highly activated, and melanized nodules around fungal hyphae (Figure 4G,H). Internal organs were preserved. On the contrary, in T11F-treated group the presence of yeast cells in the gastrointestinal tract wall was already evident at 24 h, leading to a fully fungal invasion at 48 h (Figure 4C,D). In D5A-treated larvae, big nodules with strong melanization, fat body necrosis and hemolymph clotting were observed (Figure 4E,F). For comparison, an image from an uninfected, untouched larva is shown in Figure S2.

## 3. Discussion

In the last decades there has been an increase in the number of individuals prone to fungal infections, such as immunocompromised individuals, transplant recipients and otherwise debilitated persons, while the availability of effective antifungal drugs for the treatment of mycoses has not increased at the same pace. The emergence of drug-resistant fungal strains made even more urgent the need for new antifungal therapies [41].

Among fungal pathogens, *Candida* species are the most common cause of mycoses. In particular, *C. albicans* represents a human pathobiont, being part of the commensal microbiota, and infections can arise from a derangement of the immune response and/or can be sustained or worsened by a host inflammatory milieu [42]. Since *C. albicans*-triggered diseases constitute an interesting example of an altered balance between pathogen tolerance and immune system response, targeting simultaneously infection and inflammation has been suggested as a useful antifungal strategy [43,44]. Moreover, the concept of host immunomodulation for treatment of fungal infections has gained attention in recent years, bringing the interest on immunomodulatory molecules, including antifungal peptides [41,45,46,47]. Many host defense peptides have immunoregulatory properties that appear to be essential for antifungal responses of host cells and for antifungal efficacy in vivo [14,17,47,48].

Our attention was focused on a previously described Ab-derived peptide, T11F, that showed a fungicidal activity in vitro against several pathogenic yeasts at micromolar concentration, and two of its alanine-substituted derivatives, D5A and F11A, both presenting a candidacidal activity in vitro, increased and decreased, respectively, compared to the candidacidal activity of the parent peptide. The half maximal effective concentration (EC_50_) values against the reference *C. albicans* SC5314 strain were 1.54 µmol/L for T11F, 0.39 µmol/L for D5A and 2.52 µmol/L for F11A [36]. Further studies on the structural features of these peptides revealed that T11F and D5A are characterized by the acquisition of a PPII helix conformation in aqueous solution, while F11A maintain a random coil conformation [37].

In this work, preliminarily, the direct effect on *C. albicans* cells after treatment with D5A and F11A derivatives was observed by SEM. D5A, in contrast with F11A, exhibited the same behavior as the parental T11F peptide, i.e., leakage of material from treated yeast cells and the presence of blebs on their surface were seen. F11A, instead, was not able to cause the formation of blebs and the leakage of cellular material, while gross morphological alterations of *Candida* cells were visible. These findings matched the results already obtained by confocal microscopy, confirming the hypothesis of a different interaction with yeast cells of F11A compared to T11F and D5A [37]. Even though all three peptides initially bind to *C. albicans* cells, then penetrate accumulating inside and leading to cell death, likely the different F11A conformation, namely the lack of a PPII structure, is responsible for the different effect on the membrane of the target cell, which is not so damaged as to determine an evident loss of intracellular material.

For assessing the potential therapeutic efficacy of T11F, D5A and F11A against candidiasis, we exploited the *G. mellonella* model of systemic infection. The advantages for the use of this invertebrate model include survival at mammalian temperature and the direct systemic injection of known microbial loads. Moreover, *G. mellonella* larvae allow for studying important innate immune responses against yeasts and, by recovering infected tissues, fungal pathogenic features, such as filamentation and biofilm-formation, could be observed [39,44,49].

We first checked whether the peptides themselves were toxic to the animal host. None of the examined peptides showed in vivo toxicity. We then assessed, by injecting each peptide directly into the larva hemocoel 30 min after *Candida* infection, their possible therapeutic efficacy. Interestingly, F11A derivative, with a less pronounced in vitro antifungal activity, was able to improve *G. mellonella* larvae survival to the greatest degree, likely in relation to its prominent ability to modulate the host’s immune system.

F11A, in fact, resulted the most active in triggering the larval immune response, leading to better control of fungal infection. In particular, both cytology and histology data suggested that F11A elicits the formation of tridimensional hemocyte aggregates and efficient encapsulation of *C. albicans* cells, resulting in the clearance of the pathogen. Even though histological observations showed nodule formation with melanin deposition also in T11F- and D5A-treated larvae, fully structured capsules have been detected only in F11A-treated larvae. Moreover, the peculiar presence of spherulocytes in the surroundings of granulocyte/plasmatocyte aggregates and of capsules was demonstrated by cytology and histology, respectively [40]. The involvement of spherulocytes in *G. mellonella* immune response is still unclear and the main reported function is the secretion and transportation of cuticle components. Nonetheless, an increase of spherulocytes upon a fungal trigger and their participation in nodule formation and encapsulation have been previously reported to be protective and to improve infection outcome [50,51].

In conclusion, F11A, whose direct anti-*Candida* activity has been previously demonstrated by in vitro assays, showed also an indirect anti-*Candida* activity in vivo through the stimulation of host immune cells. The double action of F11A, able to simultaneously target the fungus and the host immune system, resulted in a more efficient pathogen clearance. The obtained results confirm the potential role of Ab-derived peptides for the development of new therapeutic agents which could be endowed with both direct antifungal and immunomodulatory activity.

## 4. Materials and Methods

### 4.1. Peptide Synthesis

Synthesis and purification of T11F (TCRVDHRGLTF) and its derivatives were performed at CRIBI-Peptide Facility (University of Padua, Padua, Italy) using the fluoren-9-ylmethoxycarbonyl (Fmoc) solid-phase synthesis chemistry, as previously described [36,37,52]. Peptide purity was in a range of 80–90% as measured by analytical reverse phase HPLC.

### 4.2. SEM Studies

For SEM studies, *C. albicans* SC5314 cell suspensions, prepared as previously described [52], were incubated for 1 h in the absence (control) or presence of D5A or F11A (190 µM). 5 µL of the suspensions were placed on 25 mm^2^ glass slides, fixed with glutaraldehyde in sodium cacodylate, then prepared for microscopic examination by Philips 501 scanning electron microscope (15 kV), as previously described [52].

### 4.3. Evaluation of In Vivo Toxicity and Therapeutic Activity of the Investigated Peptides

The *G. mellonella* model was exploited for in vivo studies, according to previously described procedures [52]. For the evaluation of peptide toxicity, groups of 16 larvae (300 ± 30 mg) at their final instar stage were inoculated (10 μL/larva) with the selected peptides (15 µmol/kg). Larvae injected with 10 µL of saline were used as a control. For the evaluation of peptide therapeutic activity, groups of 16 larvae were inoculated with 10 µL of a *C. albicans* SC5314 suspension (approximately 5 × 10^5^ cells/larva) and, 30 min after, were injected with the peptides (10 µmol/kg) or saline (control). Larvae were incubated at 37 °C in the dark for 9 days and scored daily for survival. Survival curves of peptide-treated and control animals were compared by the Mantel-Cox log-rank test. A value of *p* < 0.05 was considered significant.

### 4.4. Evaluation of In Vivo Immunomodulatory Activity of the Investigated Peptides

#### 4.4.1. Hemocyte Analysis

Larvae were inoculated as above described (paragraph 4.3). Both uninfected and *C. albicans* SC5314 challenged-larvae, peptide-treated or mock-untreated (saline) were included. 24 h post-inoculation, 3 randomly selected larvae for each experimental condition were bled and the hemolymph was pooled into a microcentrifuge tube containing cold saline. For cytology, 50 μL of hemolymph was diluted 1:1 in saline prior to cyto-centrifugation at 600 rpm for 5 min. Cytospin slides were fixed with Cytofix and stained with hematoxylin and eosin. Image acquisition was performed by the NanoZoomer-XR C12000 series (Hamamatsu Photonics K.K., Tokyo, Japan). Data were confirmed in three independent experiments, and representative images are shown.

#### 4.4.2. Larval Histology

Larvae were processed for histology as previously described [49]. Briefly, three larvae per experimental condition were inoculated with 30–40 µL of buffered formalin and immersed overnight in 15 mL tubes with formalin at 4 °C. Transverse cut tissue sections were embedded in paraffin and routinely processed for conventional histopathology. Serial 4 µm sections were stained with periodic acid-Schiff stain (PAS). Image acquisition was performed by the NanoZoomer-XR C12000 series (Hamamatsu Photonics). To investigate *C. albicans* filamentation and the effect on cellular immunity in vivo, two time points were used, 24 and 48 h. Data were confirmed in three independent experiments and representative images are shown.

## Data Availability

The data supporting the findings of this study are available within the article.

## Supplementary Materials

The following are available online at https://www.mdpi.com/article/10.3390/ijms222010904/s1, Figure S1: Peptides’ toxicity in *Galleria mellonella* larvae, Figure S2: Histology of an untouched *Galleria mellonella* larva.
