# Supplementary material for: Therapeutic Effect of an Antibody-Derived Peptide in a Galleria mellonella Model of Systemic Candidiasis
Source: Int J Mol Sci. 2021 Oct 9;22(20):10904. doi: 10.3390/ijms222010904 (PMC8536055; doi:10.3390/ijms222010904)
Supplement: Supplementary file 1 [file ijms-22-10904-s001.zip › ijms-1382605-supplementary.pdf]

Therapeutic Effect of an Antibody-Derived Peptide in a *Galleria mellonella* Model of Systemic Candidiasis

Emerenziana Ottaviano [1], Elisa Borghi [1], Laura Giovati [2], Monica Falleni [1], Delfina Tosi [1], Walter Magliani [2], Giulia Morace [1], Stefania Conti [2,*] and Tecla Ciociola [2]

[1] Department of Health Sciences, University of Milan, 20142 Milan, Italy; emerenziana.ottaviano@unimi.it (E.O.); elisa.borghi@unimi.it (E.B.); monica.falleni@unimi.it (M.F.); delfina.tosi@unimi.it (D.T.); giulia.morace@unipr.it (G.M.)

[2] Department of Medicine and Surgery, University of Parma, 43126 Parma, Italy; laura.giovati@unipr.it (L.G.); walter.magliani@unipr.it (W.M.); tecla.ciociola@unipr.it (T.C.)

* Correspondence: stefania.conti@unipr.it; Tel.: +390521903492

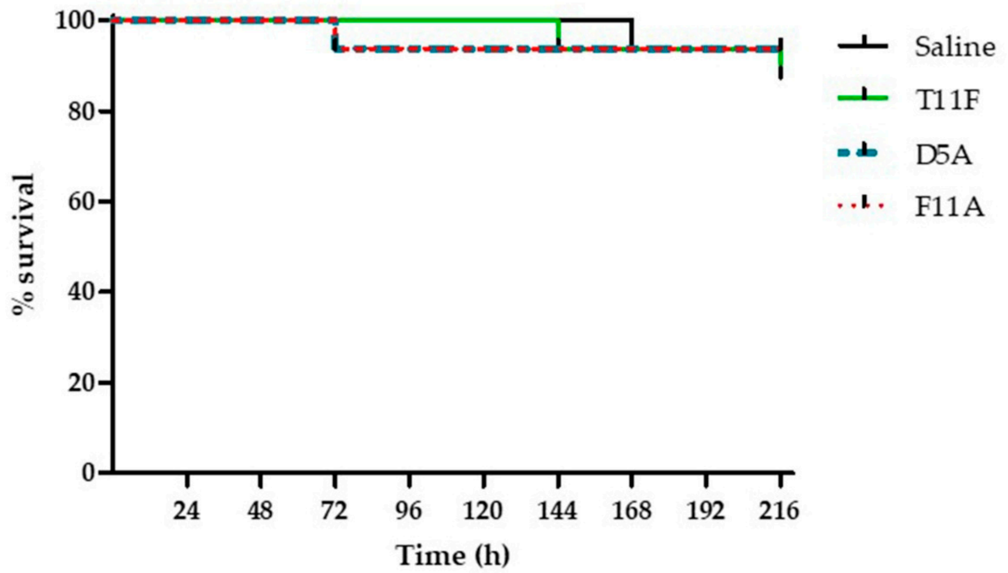

Figure S1. Peptides' toxicity in *Galleria mellonella* larvae. Larvae were inoculated with peptides (15 µmol/kg, single injection 10 µl) or saline (control group). The survival curves of treated larvae were not significantly different from that of control group, as assessed by Mantel-Cox log-rank test.

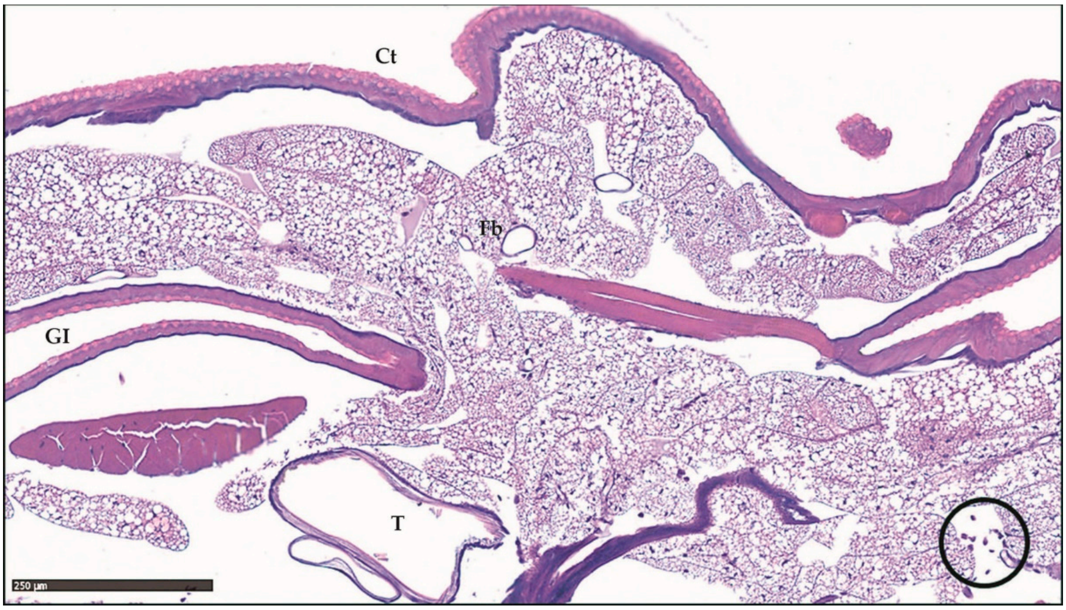

Figure S2. Histology of an untouched *Galleria mellonella* larva. Larval tissues are well preserved and only a few hemocytes can be observed in the hemolymph near the fat body (circle). Ct: cuticle; Fb: fat body; GI: gastrointestinal tract; T: trachea. Bar = 250 μm.
